# Engineering Toughness in a Brittle Vinyl Ester Resin Using Urethane Acrylate for Additive Manufacturing

**DOI:** 10.3390/polym15173501

**Published:** 2023-08-22

**Authors:** Mohanad Idrees, Heedong Yoon, Giuseppe R. Palmese, Nicolas J. Alvarez

**Affiliations:** Department of Chemical and Biological Engineering, Drexel University, 3141 Chestnut Street, Philadelphia, PA 19104, USAgrp27@drexel.edu (G.R.P.)

**Keywords:** urethane acrylate, fracture toughness, 3D printing, vat photopolymerization

## Abstract

Thermosetting polymers tend to have a stiffness–toughness trade-off due to the opposing relationship of stiffness and toughness on crosslink density. We hypothesize that engineering the polymer network, e.g., by incorporating urethane oligomers, we can improve the toughness by introducing variations in crosslink density. In this work, we show that a brittle methacrylated Bis-GMA resin (known as DA2) is toughened by adding a commercial urethane acrylate resin (known as Tenacious) in different proportions. The formulations are 3D printed using a vat photopolymerization technique, and their mechanical, thermal, and fracture properties are investigated. Our results show that a significant amount of Tenacious 60% *w*/*w* is required to produce parts with improved toughness. However, mechanical properties drop when the Tenacious amount is higher than 60% *w*/*w*. Overall, our results show that optimizing the amount of urethane acrylate can improve toughness without significantly sacrificing mechanical properties. In fact, the results show that synergistic effects in modulus and strength exist at specific blend concentrations.

## 1. Introduction

Thermosetting resin parts are traditionally produced via molding techniques or cured part machining [1,2]. The advent of additive manufacturing techniques, such as vat photopolymerization, have enabled the use of light to make thermoset parts without a mold. This process, also known as 3D printing, allows for the creation of complex and intricate parts that would be difficult or impossible to produce using traditional methods [3,4,5]. The most commonly used thermoset resins in 3D printing include acrylate and methacrylate monomer/oligomers, which offer a wide range of mechanical properties. However, the polymerization process results in high crosslink density networks and thus brittle parts, limiting their suitability for load-bearing applications [6,7,8,9,10]. For example, Jianwei et al. developed methacrylated BisGMA photo resin (DA2) with a high glass transition temperature and stiffness. However, this resin exhibited an extremely low toughness (0.058 kJ/m^2^) [6], which significantly limits its applicability in engineering applications.

There are several approaches to improving the mechanical properties and fracture resistance of brittle resins. One method is the incorporation of inorganic fillers, such as fumed silica and calcium carbonate [10]. Another approach is using tough particle fillers, such as rubber particles or liquid rubber, which form phase-separated domains that improve energy dissipation through plastic deformation, shear yielding, and cavitation of the phase-separated domains. These approaches improve toughness but at the cost of reduced strength and stiffness and/or result in a low glass transition temperature [7,10]. A combination of a rubbery material and stiff filler, such as graphene oxide and carbon nanotubes, has also been used to enhance the performance of thermosetting resins. This approach is referred to as “hybrid toughening”, and has been shown to increase toughness by an order of magnitude [11,12,13]. A third method is using a chain transfer agent to regulate the formation of a polymer network, such as through an addition fragmentation chain transfer (AFCT) mechanism [14]. Overall, high toughness is either due to the filler/second phase or the network structure. Certain thermosetting resins exhibit a high toughness and ductile behaviour due to either their low crosslink density or their molecular structure, such as urethane resins, but their strength and stiffness fall short for structural engineering applications [15,16,17].

Urethane acrylate resins are widely used in coating applications due to their strong adhesion, resilience, and high resistance to impact [18]. They are synthesized through reacting diisocyanate and polyol functional groups. The properties of urethanes are engineered by adjusting the length and structure of the bifunctional alcohol used in its synthesis. For example, using long aliphatic polyol results in a product with high elongation and toughness, and a short aromatic polyol produces a rigid polymer [19]. Several soft and flexible urethane resins for 3D printing are commercially available under different brand names, such as Tenacious produced by Siraya Tech [20]. However, their low stiffness and Tg make their applicability in engineering applications very limited.

Blending urethane acrylate with brittle epoxy or epoxy acrylate, such as DGEBA, can result in a resin with desirable properties. Several studies have reported this, including Hua and Hu’s investigation of the compatibility of epoxy resin and urethane acrylate, which showed an improvement in tensile elongation and impact strength with increasing urethane content [21]. Similarly, Xu et al. blended hyberbranched urethane acrylate with epoxy acrylate at a low to moderate percentage (5–40%) and found that as little as 10% of urethane acrylate increased fracture toughness by a factor of 1.7, and an improvement in strength was also observed without any change in modulus. The toughening mechanism is thought to be due to the reduction of the volume of rigid epoxy segments [22]. Additionally, a synergistic effect in flexural properties has been reported when epoxy (DGEBA) and urethane dimethacrylate (UDMA) are mixed at 30% and 70%, respectively, which was attributed to the DGEBA segmental motion being hindered by UDMA [23].

This paper aims to improve DA2 toughness and enable its utilization in advanced applications by incorporating soluble and co-reacting urethane acrylate precursors (via a commercial resin called Tenacious). We focus our attention on tensile, flexural, and fracture toughness properties. Mechanical properties are measured as a function of the ratio of Tenacious in DA2 Resin between 20–80% *w*/*w*. The results clearly show that the mechanical and fracture properties have unexpected non-monotonic behavior with the weight fraction of the blends. Note that the observed trends in fracture toughness are for single network polymers, whereby no phase separation or secondary phase is necessary to increase fracture toughness, which is the common mechanism in epoxy systems.

## 2. Materials and Methods

Bisphenol A glycerolate dimethacrylate (Bis-GMA), ethoxylated Bisphenol A dimethacrylate (Bis-EMA), and 1,6-hexanediol dimethacrylate (HDDMA) were purchased from Esstech, Inc. (Essington, PA, USA). Phenylbis (2,4,6 trimethylbenzoyl) phosphine oxide (PPO) were purchased from Sigma Aldrich, inc (St. Louis, MO, USA). DA2 Resin was formulated by mixing Bis-GMA, Bis-EMA, and HDMMA at ratios of 3:3:2, followed by adding PPO at 0.7% *w*/*w*. A detailed description of DA2 resin formulation and characterization was previously published by Jianwei et al. [6]. A Urethane acrylate commercial resin (Tenacious) was purchased from Siraya tech (San Gabriel, CA, USA). The resin is a mixture of Bisphenol A ethoxylate diacrylate and urethane acrylate.

**Figure 1:**
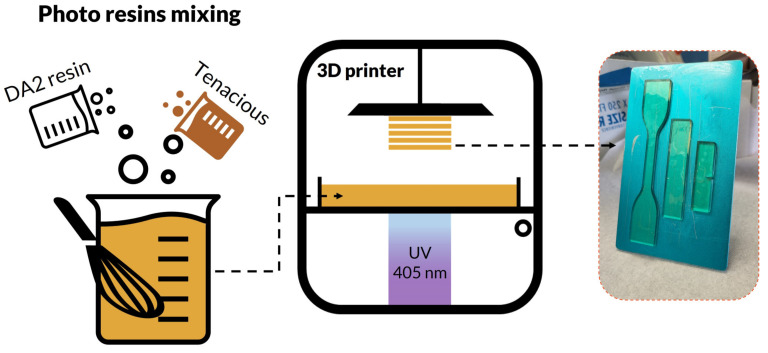
Schematic of photo resin formulation and 3D printing.

Figure 1 shows a schematic of the resin mixing and its 3D printing process. Tenacious and DA2 are mixed at different proportions, and the percentage of Tenacious resin is varied from 20 to 80% *w*/*w*. The resins are mixed for two hours using a dynamic mixer at a speed of 2000 rpm. The mixing process is followed by degassing in a vacuum oven to remove entrapped air. Resins viscosity was measured using TA Instruments AR2000 rheometer (New Castle, DE, USA) at 25 °C under steady state flow using a cone-plate geometry and shear rate range of 0.001 s^−1^ to 100 s^−1^, the viscosity in shear rate range of 0.1–100 s^−1^ is reported. Three samples were tested, and average viscosity was obtained. A vat photopolymerization 3D printer (Anycubic Photon Mono SE, Guangdong, China) was used for printing test coupons. The printer delivers a power density of 3.0 mW/cm^2^, enabling faster print time. Test coupon CAD models were sliced for 100 µm layer thickness, and 20 s exposure was used for three bottom layers to enhance the part adhesion to the building platform; the remaining layers’ exposure time was set to 7 s. The printed parts were carefully removed from the build platform using a razor blade, wiped from the excess resin, and postcured in a UV oven (Formlabs Inc., Somerville, MA, USA) for two hours at 75 °C. Finally, specimens were polished to eliminate surface defects.

To determine the degree of conversion, both neat resins and post-cured samples were analyzed using mid-infrared (Mid-IR) spectroscopy. The Mid-IR spectra were obtained with a Thermo Nicolet Nexus 870 FT-IR spectrometer, equipped with a deuterated triglycine sulfate (DTGS) detector in the range of 650–4000 cm^−1^ (Thermo Fisher Scientific, Waltham, MA, USA). Spectra were collected using 120 scans at a resolution of 4 cm^−1^. The typical peaks used for the evaluation of methacrylate double bound conversion (at 1608 and 1636 cm^−1^) exhibited shifts and overlaps upon cure. Thus, an alternative approach is used. The conversion was evaluated in accordance with the methodology described in [24] by using the peak at 1320 cm^−1^ as a reactive peak in combination with the peak at ~1720 cm^−1^ as a reference peak. The conversion was determined using the following equation:

(1) $$\propto =1-\frac{{\left(\frac{h_{1320\;{\mathrm{cm}}^{-1}}}{h_{1720\;{\mathrm{cm}}^{-1}}}\right)}_{}}{{\left(\frac{h_{1320\;{\mathrm{cm}}^{-1}}}{h_{1720\;{\mathrm{cm}}^{-1}}}\right)}_{0}}$$

The printed formulations were characterized using a dynamic mechanical analyzer (DMA) (Q800 DMA, TA Instruments, New Castle, DE, USA) to investigate their thermomechanical performance, e.g., glass transition temperature and the effect of the two resin ratios on the polymer network structure. Specimens of~35 mm × 12.7 mm × 3.0 mm were cut from flexural specimens and tested in a single cantilever mode using 1 Hz frequency, an amplitude of 10 µm, and a heating rate of 2 °C/min. All mechanical properties (tensile, flexure, and fracture toughness) testing was carried out on a servo-hydraulic Instron apparatus with the appropriate load cell (Instron, Norwood, MA, USA). Tensile properties were measured according to the ASTM D638-14 standard. At least five dog bones (type IV) were tested. An extensometer was used to accurately measure the strain, and strain data in the 0.05–0.25% range were used to calculate the tensile modulus. Resins’ flexural properties were measured also in Instron as per ASTM D790. Rectangular bars of sizes 120 mm × 12.7 mm × 3.0 mm were tested in a three-point bending setup and the span-to-thickness ratio of 16 ± 1. Fracture toughness K_IC_ and strain energy release rate per unit area G_IC_ were measured as per ASTM D5045-14. Single-edge-notch-bend (SENB) specimens were pre-cracked using a razor blade, and 5–10 specimens of each set were tested at a crosshead speed of 10 mm/ min. The SEM micrographs of the SENB fracture surface were obtained using Quanta 600 ESEM.

## 3. Results and Discussion

Table 1 shows the viscosity of four resin blends with Tenacious fractions between 20–80% *w*/*w*. Note that it is not serendipitous that the two pure resins have similar viscosity. They are designed for use in vat photopolymerization, which works well with resin viscosity below 500 cP. Above this viscosity, it is difficult for bubbles, which are formed during the motion of the building platform, to escape during operation. Low viscosity ensures that resin will flow in a reasonable timescale (i.e., a few seconds) to fill previously cured areas [15,16]. For all blends, the viscosity was found to be within the range of 507–533 cP, which is higher, but relatively similar, to the viscosity of the individual resins. Thus, these blends are suitable for vat photopolymerization.

**Figure 2:**
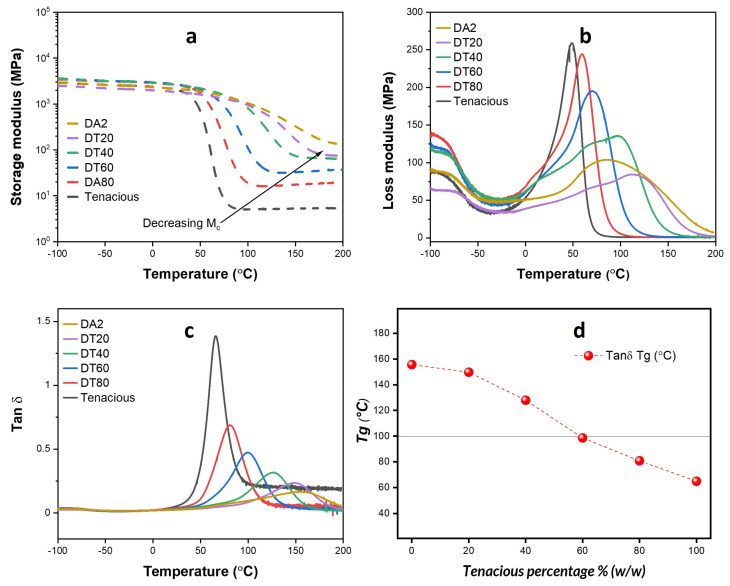
Thermomechanical properties: (**a**) storage modulus of different blends; (**b**) loss modulus; (**c**) Tan δ; (**d**) summary of glass transition temperature for different blends.

DMA results for all samples are presented in Figure 2. Figure 2a shows the storage modulus as a function of temperature. All resins have a room temperature storage modulus higher than 2000 MPa (2.0 GPa). The glassy-rubbery transition regime for DA2 shows a gradual decrease in storage modulus, while Tenacious shows an approximately two orders of magnitude drop in modulus over a 20 °C drop in temperature. Wilson et al. also observed this very fast drop in modulus in aliphatic urethanes. They hypothesized that the fast drop is due to a monodisperse molecular weight between crosslinks [25]. Pure DA2 showed a short rubbery plateau, and the magnitude of rubbery modulus decreased with the increasing Tenacious percentage in other resins. Thus, as expected, the crosslink density of the blend decreases with an increasing percentage of Tenacious.

Figure 2b shows the loss modulus as a function of temperature. It is well understood that the peak in loss modulus provides information about the structure of the polymer network and energy dissipation, e.g., a lower peak in loss modulus indicates a more brittle material. The loss modulus is very broad for pure DA2 and blends with a high DA2 fraction, indicating a heterogeneous network structure, which is common in acrylate-based polymers [10,26]. The magnitude of the peak in loss modulus increases and shifts to the left with increasing Tenacious content, indicating a higher energy dissipation and a decrease in glass transition temperature. This result is further exemplified in Figure 2c, which shows the tan δ as a function of temperature. The magnitude of the peak in tan δ is proportional to the fraction of Tenacious, indicating more dissipative behavior with increasing Tenacious fraction. One interesting observation is that only Tenacious shows a steady high tan δ after the glassy-rubbery transition, which indicates the presence of a significant number of dangling chains [25].

Figure 2d shows the measured Tg (based on tan δ) as a function of the Tenacious fraction. One unexpected result is the nonlinear dependence of Tg on the Tenacious fraction. In other words, the blends do not follow a simple rule of mixtures. The DA2 and DT20 show significantly higher Tg than the other blends. This result suggests that a dilute presence of urethane does not significantly dilute the DA2 polymer network. It is only above a fraction of 50% Tenacious that we see a significant decrease in glass transition temperature.

**Figure 3:**
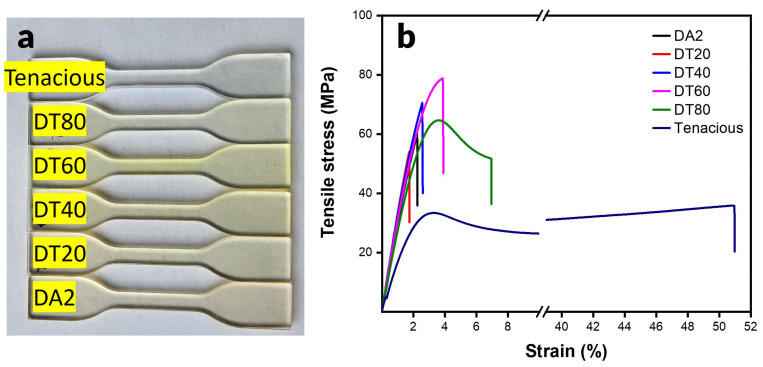
Tensile specimens and properties: (**a**) 3D printed dogbone specimens; (**b**) representative tensile stress strain curves of DA2, Tenacious, and their blends.

High tensile modulus and strength are highly desired for load-bearing applications. A snapshot of 3D printed dog bone specimens are shown in Figure 3a. The print quality is nearly the same for all of the specimens. However, there is a color difference due to monomer composition. Note that there is no observed phase separation in any of the blended compositions. Figure 3b shows the stress-strain curves for all samples. The representative stress-strain curve shows that Tenacious exhibits a high elongation at break up to ~50%. This is in contrast to pure DA2 and blends with large DA2 fractions (DT20 and DT40), which showed a typical brittle fracture with a linear stress strain curve and abrupt failure. When Tenacious percentage is increased to 60% (DT60), we start to observe a transition to a ductile behavior with a defined yield point and more than a 100% increase in the strain at break over DA2. The 80% Tenacious blend shows a typical ductile failure with yield point, plastic deformation regime, and more than a twofold increase in the strain at break compared to DA2. The results indicate that a minimum fraction of the urethane acrylate is required to introduce yielding behavior and plastic deformation to the network. The available literature does not specify a certain amount of urethane for improved flexibility. In fact, the amount of urethane required to produce a noticeable increase in elongation is variable due to the variable molecular structures of urethane and the epoxy systems used [21,22,27].

**Figure 4:**
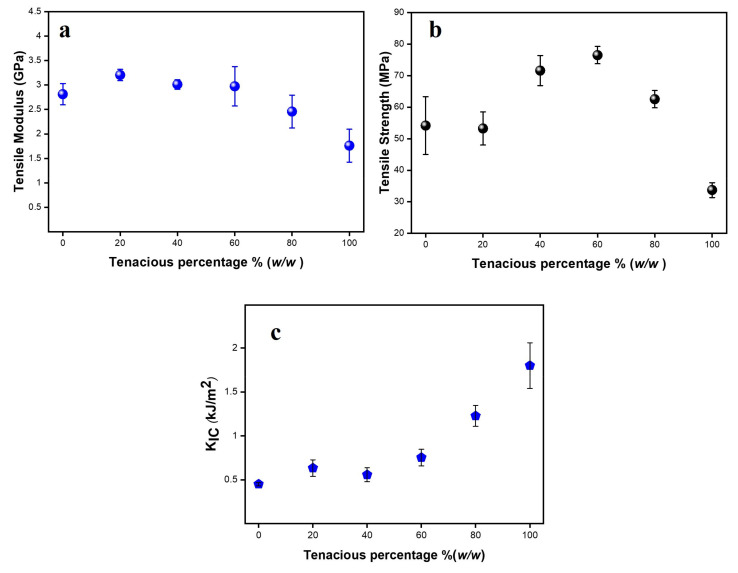
(**a**) Tensile modulus vs. Tenacious percentage; (**b**) Tensile strength vs. Tenacious percentage; (**c**) Fracture toughness of DA2, Tenacious, and their blends.

The tensile and fracture properties of Tenacious, DA2, and their mixtures are tabulated in Table 2, and are also shown in Figure 4a–c. The tensile modulus and strength of pure DA2 are significantly higher than pure Tenacious. In typical blends, one would observe that mechanical properties follow a rule of mixtures between the two pure components, e.g., a modulus of the blends between 1.7 and 2.8 GPa. However, in Figure 4a, we see that the modulus of DA2/Tenacious blends shows no reduction in modulus up to 60% Tenacious. More importantly, DT20 has the highest modulus (3.2 GPa), which indicates a 15% increase in modulus over pure DA2. Note that this increase in modulus is not supported by an increase in crosslink density, as discussed above. At present, we do not have an explanation for this trend, but it could be attributed to the interaction of urethane and BisGMA resulting in a restricted motion. A similar explanation was offered for urethane and epoxy blends [23].

The non-monotonic result in modulus is also observed in the tensile strength (Figure 4b). The tensile strength of DA2 and DT20 was moderate (in the range of 50–60 MPa). However, blends with moderate DA2 (DT40 and DT60) show exceptional tensile strength greater than 70 MPa. The improvement in tensile strength might be due to their network structure and improved toughness, as the less dense networks tend to be more flexible, resulting in increased toughness and less vulnerability to defects and premature failure. Overall, systems with higher contents of DA2 are brittle. Importantly, doping Tenacious with 20% DA2 (DT80) results in an improved strength over Tenacious, e.g., DT80 shows strength almost twice that of pure Tenacious. Overall, the strength of blended resins was higher than DA2.

The tensile strength and the tensile behavior of the blends might be better understood by trends in fracture toughness. The fracture toughness (K_IC_) and strain energy release rate per unit area (G_IC_) are tabulated in Table 2; the dependence of K_IC_ on Tenacious percentage is shown in Figure 4c. Resins with low tenacious content e.g., DT20 and DT40, did not show a significant improvement in G_IC_ compared to pure DA2, e.g., ~0.1 kJ/m^2^, which is considered low and justifies the brittle tensile failure observed in Figure 3b. However, as Tenacious percentage increases, we observe a more pronounced toughness improvement: for example, a two times increase in G_IC_ was achieved for 60% Tenacious, and a nearly ninefold increase in G_IC_ for DT80. Note that these toughness values are equivalent to some toughened vinyl ester resins [28]. The improved toughness of DT80 and DT60 coincides with their improved strength. In conclusion, K_IC_ and G_IC_ are insensitive to low Tenacious percentages (20 and 40%), but they are influenced strongly at moderate to high Tenacious percentages (60 and 80%), signifying a polymer network whose fracture is controlled by urethane.

**Figure 5:**
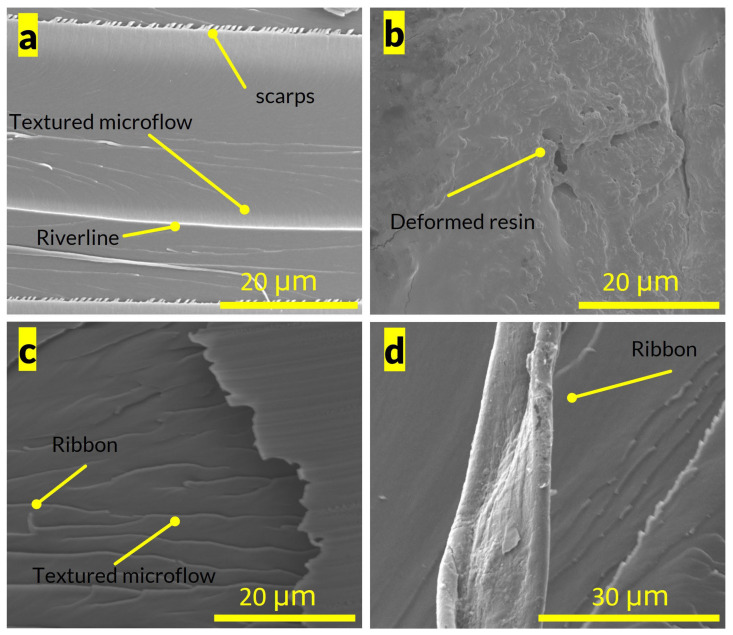
Fracture surface morphology-SEM, (**a**) DA2, (**b**) Tenacious, (**c**) DT60, (**d**) DT80.

Figure 5 shows SEM images of the fracture surface morphology for the SENB specimens. Figure 5a (Top left) shows the DA2 surface, which exhibits characteristic features of brittle failure, as evidenced by the overall smooth texture with river-line and textured microflow patterns [29]. Conversely, Figure 5b shows the fracture surface of Tenacious, which exhibits granular/dimpled morphology typical of tough resins. Figure 5c,d shows the fracture surface of blended resins, DT60 and DT80, which exhibit behavior different from DA2 and Tenacious, with a relatively coarse texture (Figure 5c,d). Notably, features such as ribbons become more prominent with increasing Tenacious content. However, no dimples are observed. Overall, we observe unique fracture patterns for each composition of resin. The SEM images confirm that there is no observed phase separation in any of the resin compositions on the nanoscale. Therefore, the enhanced toughness can solely be ascribed to the unique chemistries of the resins and their respective network properties.

**Figure 6:**
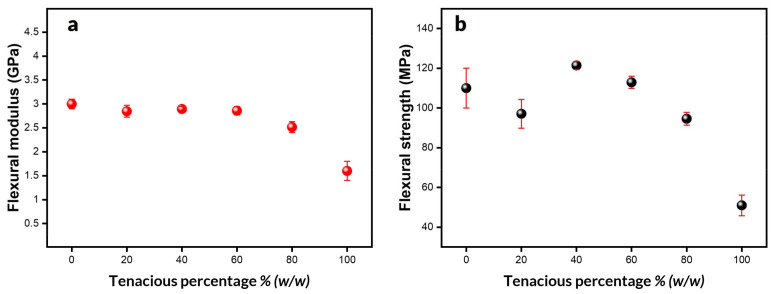
Flexural properties: (**a**) flexural modulus; (**b**) flexural strength.

In addition to Tensile properties, flexural properties are very important in functional parts subjected to bending during service. Furthermore, flexural properties are less sensitive to defects compared to tensile properties. Figure 6a,b summarize flexural properties as a function of Tenacious percentage. As with tensile properties, the flexural modulus is relatively constant, up to 60% Tenacious, and decreases at higher concentrations. Moreover, the flexural strength of blends goes through a maximum, similar to the tensile properties. However, the flexural strength values are significantly higher. DA2 and DT20 exhibited a lower average strength value compared to DT60 and DT40. However, it is essential to mention that they show a more significant standard deviation, which we hypothesize is due to their brittleness. In general, all blends showed a flexural strength of 100 MPa or higher. DT40 shows the highest strength (121 MPa). Note that this is one of the highest strength values reported for photo resins, which are usually in a range of 50–120 MPa [15,30,31,32]. It is worth noting that the samples with high Tenacious content do not fail during testing. In these cases, the flexural strength was measured at the maximum strain recommended by the ASTM standard (maximum of 0.05 mm/mm). In general, the flexural strength values for all blends were significantly higher than their tensile strength values, by about 50%. This difference can be attributed to the localized nature of flexural properties and their lower sensitivity to defects, which allows them to withstand heavier loads.

**Figure 7:**
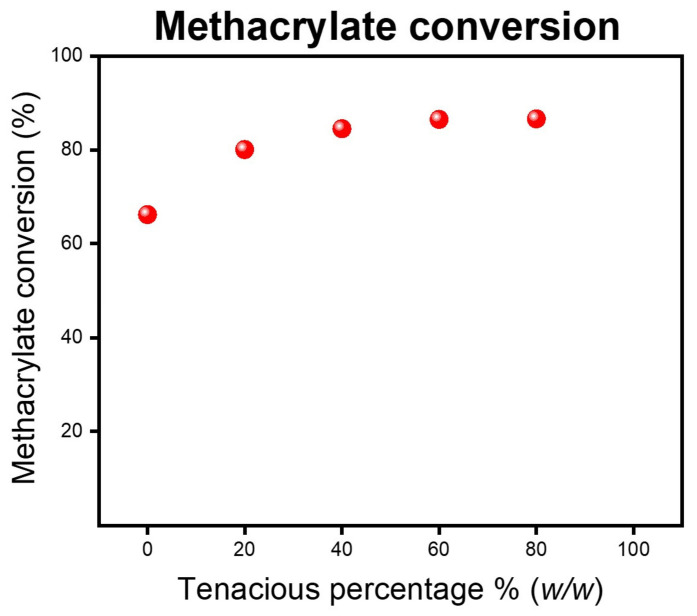
Post-cured resins methacrylate conversion as a function of Tenacious percentage in the resin.

The blended resins consist of a combination of acrylate and methacrylate functional groups. DA2 has primarily methacrylate functional groups, whereas Tenacious has acrylate groups. Figure 7 shows the methacrylate conversion for post-cured samples. We see that the addition of Tenacious monotonically improves the degree of methacrylate conversion, but above 40% *w*/*w* of Tenacious, there is only a slight increase in methacrylate conversion. We also looked at the acrylate conversion using the peak at 1407 cm^−1^ pertaining to the acrylate groups on Tenacious in the blends. This peak is completely missing from the post-cured spectra (see Supplementary Materials, Figures S2–S6). This suggests nearly full conversion of all acrylate groups in the blends, which is expected given the higher reactivity of acrylates to methacrylates. One might hypothesize that the mechanical properties observed in Figure 4 and Figure 6 are proportional to the degree of methacrylate conversion. However, the non-monotonic mechanical results in Figure 4 and Figure 6 clearly indicate that there is a synergistic effect of the blend that cannot simply be correlated to the methacrylate conversion. Spectra of pure resins and cured parts are included in the Supplementary Materials Figures S1–S6.

## 4. Conclusions

This paper studies resin blends for use in additive manufacturing processes, specifically in vat photopolymerization. These blends consist of two resins, Tenacious and DA2, which have been optimized for low viscosity to prevent bubble formation during 3D printing. The paper characterizes the 3D printed formulations using dynamic mechanical analysis to investigate their thermomechanical performance, including glass transition temperature and the effect of the resin ratios on the polymer network structure. The results show that the glass transition temperature decreases with increasing Tenacious content and that blends with higher DA2 content exhibit thermomechanical performance similar to pure DA2. We clearly show that there are synergistic mechanical properties that result from the blending of these two resins. For example, there is a maximum in tensile modulus and strength at an intermediate fraction of Tenacious.

Overall, our results demonstrate that blending a Methacrylated BisGMA resin with a urethane based acrylate resin produces parts with improved toughness without compromising strength and stiffness. Furthermore, it demonstrates increased toughening of single network polymers without the use of fillers or phase separated rubbery domains, commonly employed to toughen epoxy resin systems. The blend of Methacrylate resin and urethane acrylate resin shows promise as a material that can provide a good balance of toughness, strength, and modulus for vat photopolymerization additive manufacturing applications. Similar synergistic effects may result from other blends of urethane and methacrylate resins. However, note that the resins studied here are complex mixtures of five monomers, and it is not clear whether the same trends will be observed for simpler blends. More work is needed to understand the molecular origin of these synergistic trends in mechanical properties. In future work, we plan to use these resins as a matrix in glass fiber composites.

## Figures and Tables

**Table 1:** Resins compositions and viscosity.

Resin Name	DA2 % (*w*/*w*)	Tenacious % (*w*/*w*)	Resin Viscosity (cP)
DA2	100	0	490 ± 50 [6]
DT20	80	20	524 ± 7
DT40	60	40	521 ± 8
DT60	40	60	533 ± 45
DT80	20	80	507 ± 24
Tenacious	0	100	503 ± 85

**Table 2:** Resins tensile and fracture properties.

Blend	Tensile Modulus (GPa)	Tensile Strength (MPa)	Strain at Break (%)	K_IC_ (MPa.m^0.5^)	G_IC_ (kJ/m^2^)
DA2	2.8 ± 0.2	54.1 ± 9.2	1.9 ± 0.5	0.45 ± 0.02	0.058
DT20	3.2 ± 0.1	53.2 ± 5.2	1.7 ± 0.2	0.63 ± 0.09	0.128
DT40	3.0 ± 0.1	71.6 ± 4.8	2.6 ± 0.2	0.55 ± 0.08	0.097
DT60	2.9 ± 0.4	76.5 ± 2.7	4.1 ± 1.0	0.75 ± 0.09	0.18
DT80	2.5 ± 0.3	62.5 ± 2.7	6.8 ± 2.5	1.22 ± 0.12	0.544
Tenacious	1.7 ± 0.3	33.7 ± 2.3	42.0 ± 8.0	1.8 ± 0.26	1.58

## Data Availability

Data is available upon request.

## Supplementary Materials

The following supporting information can be downloaded at: https://www.mdpi.com/article/10.3390/polym15173501/s1, Figures S1–S6: FTIR spectra of neat resins and the cured resin parts.
