# Supplementary material for: Engineering Toughness in a Brittle Vinyl Ester Resin Using Urethane Acrylate for Additive Manufacturing
Source: Polymers (Basel). 2023 Aug 22;15(17):3501. doi: 10.3390/polym15173501 (PMC10490117; doi:10.3390/polym15173501)
Supplement: Supplementary file 1 [file polymers-15-03501-s001.zip › polymers-2543119-supplementary.pdf]

Supporting information

Figure S1 through S6 show the mid-IR spectra of the neat resins and also the spectra after UV post cure

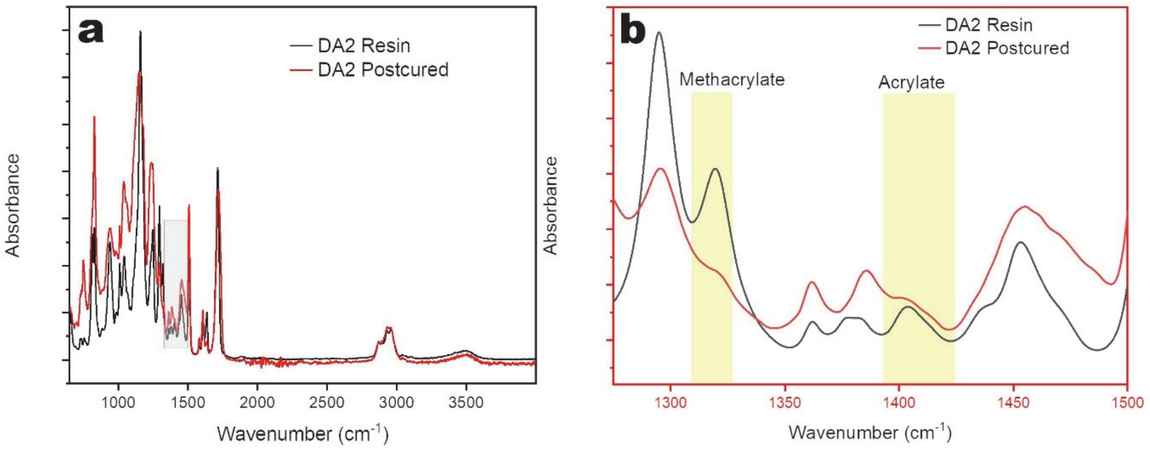

Figure S 1 (a) Full spectra of DA2 Resin and Postcured DA2, (b) Enlarged spectra of DA2 Resin and Postcured DA2

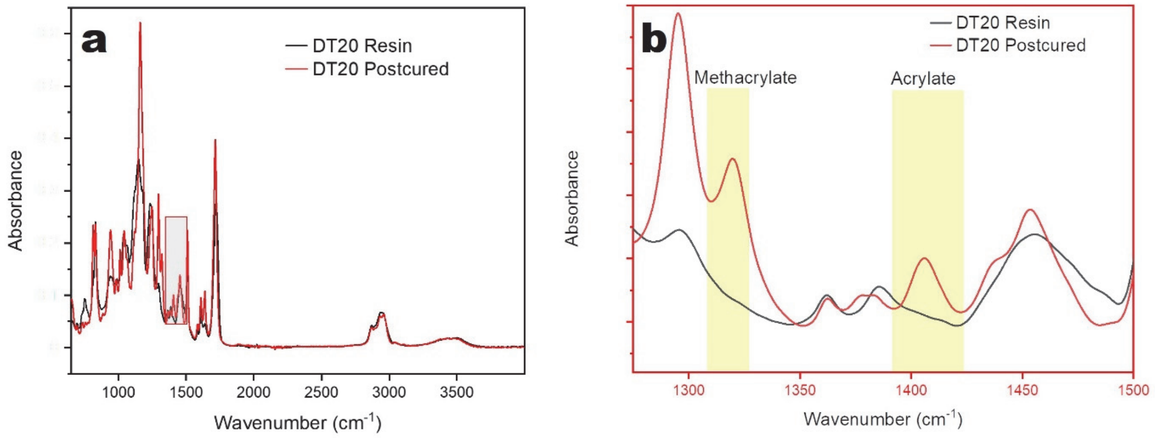

Figure S 2 (a) Full spectra of DT20 Resin and Postcured DT20, (b) Enlarged spectra of DT20 Resin and Postcured DT20

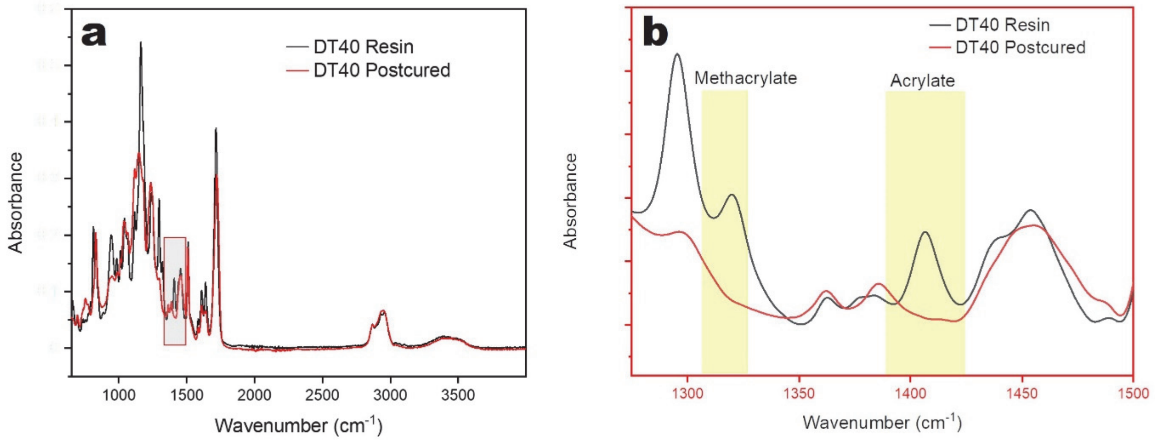

Figure S 3 (a) Full spectra of DT40 Resin and Postcured DT40, (b) Enlarged spectra of DT40 Resin and Postcured DT40

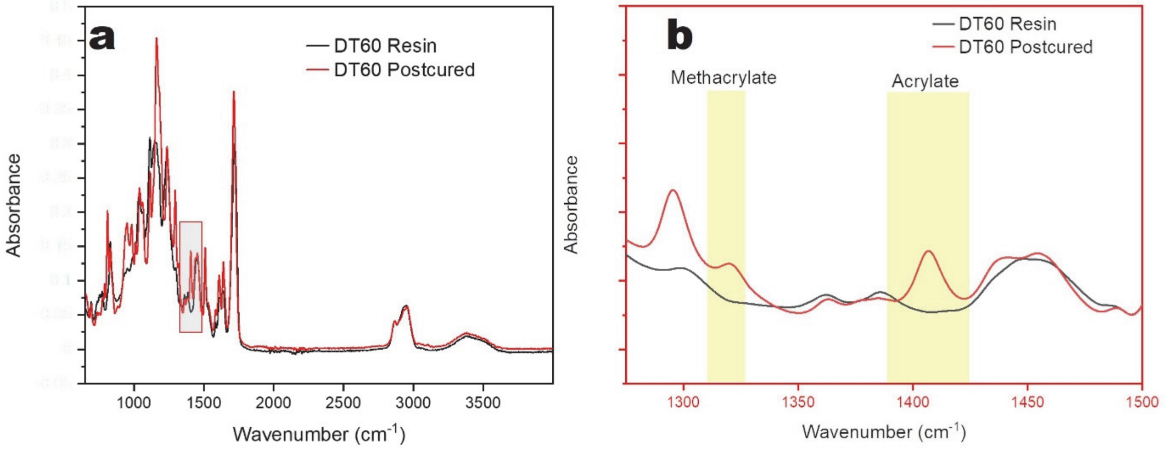

Figure S 4 (a) Full spectra of DT60 Resin and Postcured DT60, (b) Enlarged spectra of DT60 Resin and Postcured DT60

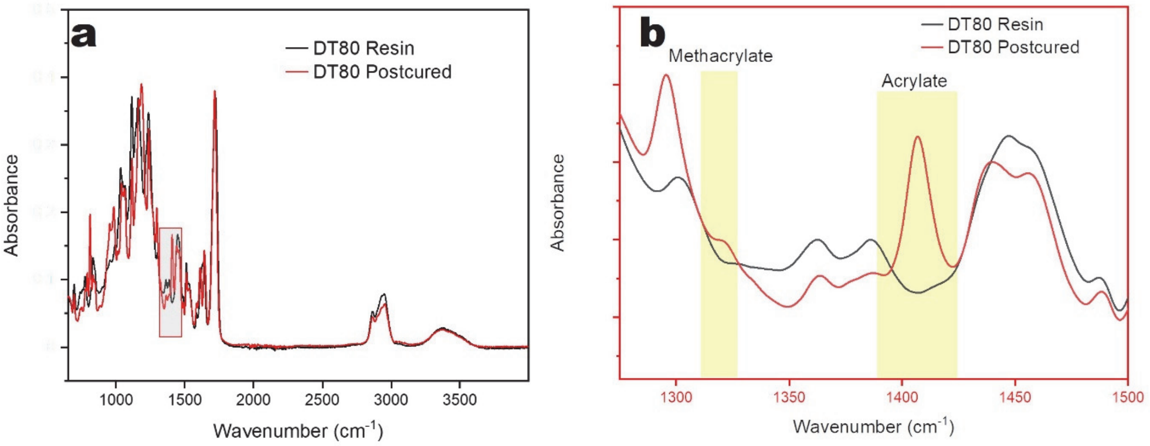

Figure S 5 (a) Full spectra of DT80 Resin and Postcured DT80, (b) Enlarged spectra of DT80 Resin and Postcured DT80

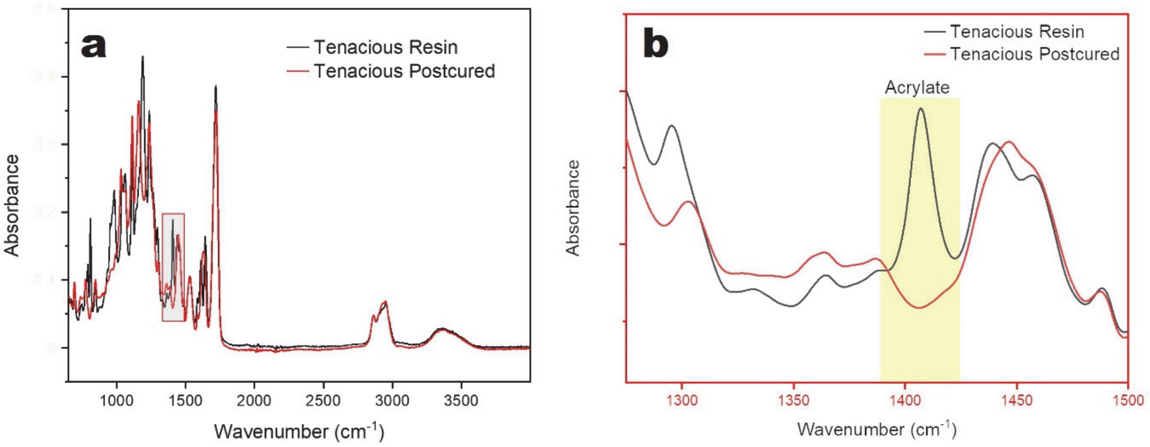

Figure S 6 Full spectra of Tenacious Resin and Postcured Tenacious, (b) Enlarged spectra of Tenacious Resin and Postcured Tenacious
